# Nitrogen fertiliser value of biogas slurry and cattle manure for maize (*Zea mays* L.) production

**DOI:** 10.1016/j.heliyon.2021.e07077

**Published:** 2021-05-22

**Authors:** T. Mdlambuzi, P. Muchaonyerwa, M. Tsubo, M.E. Moshia

**Affiliations:** a South African Sugarcane Research Institute, Private Bag X02, Mount Edgecombe, 4300, South Africa; b School of Agricultural, Earth and Environmental Sciences, Soil Science Discipline University of KwaZulu-Natal, Private Bag X01, Scottsville, 3209, South Africa; c Arid Land Research Center, Tottori University, 1390 Hamasaka, Tottori, 680-0001, Japan; d Department of Agronomy, Faculty of Science and Agriculture, University of Fort Hare, Private Bag X1314, Alice, 5700, South Africa; e Agricultural Research Council – Soil, Climate and Water, Private Bag X79, Pretoria, 0001, South Africa

**Keywords:** Maize yield, Nutrient uptake, Phosphorus, Potassium

## Abstract

Recovery of nutrients from biogas slurry (BGS) as a soil amendment, on low input smallholder farms in sub-Saharan Africa, could improve agricultural production and minimize contribution of the agroecosystems to CO_2_ emissions. Comparative effects of BGS and cattle manure (CM) on maize dry matter, grain yield, uptake of nitrogen (N), phosphorus (P) and potassium (K), and soil total N, extractable P and exchangeable K after harvest were studied, relative to chemical fertiliser (CF). The field experiment was conducted in the 2016/2017 and 2017/2018 growing seasons and was arranged in a randomized complete block design replicated four times with (i) BGS, (ii) CM and (iii) CF as the treatments. Each treatment was applied at 40, 80 and 120 kg Nha^−1^. Additional P was added to BGS and CM to have the same added P as in the CF treatments. The CM treatment had higher dry matter than both BGS and CF in both seasons at each N rate. Maize grain yield from CF treatment was higher than the two organic fertilisers at each N rate, while the BGS treatment had higher grain yield than CM except at 40 kg Nha^−1^. When applied at the same N rate, BGS resulted in lower P and K than CF, and had higher extractable P with lower exchangeable K when compared with CM. The findings imply that while BGS provided nutrients, it resulted in lower maize dry matter than CM and lower grain yield than CF, but raised total N and available P, over time.

## 1 Introduction

The decline in soil fertility associated with agricultural intensification and continuous cultivation, without replenishing nutrients, is a major problem for the agricultural sector (Gurung, 1997; Jones et al., 2013). Inadequate nutrient supply and poor soil quality are major constraints in low input agriculture (Khan et al., 2012). Intensive application of CF on smallholder farms is limited by their high costs, while on highly resourced farms intensive application has led to several issues such as pollution of water with nitrate and phosphate and loss of soil carbon (Nkoa, 2014). Problems associated with low soil fertility and nutrient management could be resolved by amendments with organic waste materials that positively influence soil fertility and crop productivity. Organic wastes need to be utilized to avoid waste or loss of nutrients to the environment (Smith et al., 2014). A vast range of organic fertilisers such as manures and compost have been considered as options to improve soil fertility. Instead of direct soil application, manure can also be used to produce biogas for energy, with the potential benefit of an organic fertiliser (biogas slurry) as a by-product from the same waste. Biogas slurry from anaerobic digestion of various organic wastes through the biogas technology has received great attention worldwide (Paul and Beauchamp, 1993; Islam, 2006; Weiland, 2010; Abubaker et al., 2012; Smith et al., 2014; Nyang'au et al., 2016).

Biogas slurry is reported to be rich in macro- and micronutrients in readily available forms, which are essential for plant growth and development (Ward et al., 2008; Smith et al., 2014; Kumar et al., 2015; Cao et al., 2016). The high nutrient composition of BGS suggests that it has the potential to be used as an organic fertiliser (Odlare et al., 2011) while providing a cheaper and safer alternative source of nutrients compared to chemical fertilisers (CF) (Khan et al., 2012). Biogas slurry has the potential to improve crop nitrogen (N) uptake, growth and yields, and adds the necessary organic carbon (OC) and improve soil quality (Ernst et al., 2008; Bachmann et al., 2011; Galvez et al., 2012). Furthermore, the nutrient cycling and liming effects of BGS could improve soil quality and crop yields, with insignificant negative effects (Nyang'au et al., 2016). The use of BGS as a nutrient source could reduce the need for the use of CF, reducing fertiliser costs, especially for farmers in the smallholder sector in the vicinity of biogas plants (Kumar et al., 2015). Several studies have reported that anaerobically digested cattle and pig slurry, which are sometimes referred to as BGS could improve structure, water-holding capacity and overall fertility of the soil, increase crop yields (Paul and Beauchamp, 1993; Nasir et al., 2012; Nasir et al., 2015; Malav et al., 2015a,b; Zheng et al., 2016) compared to CF and other organic composts. Several studies have reported that effects of CF and BGS are comparable in terms of crop yields (Dauden and Quilez, 2004; Nasir et al., 2012; Kumar et al., 2015).

A variety of BGS produced from different production systems contained 0.5–2.5% N, 0.5–1.9% phosphorus (P) and 0.6–2.2% potassium (K) (Khandeiwal and Mahdi, 1986; Demont et al., 1990; Surendra et al., 2014; Kumar et al., 2015). The variation in the nutrient composition of BGS could depend on the chemical composition of the feedstock and biogas production conditions used. For example, solid animal manure N, P and K could range between 0.4-0.8%, 0.6–0.8% and 0.5–0.7%, respectively, depending on feed and storage conditions (Surendra et al., 2014). Cattle manure (CM) is the most common organic waste on resource-poor farms in sub-Saharan Africa, and its nutrient composition is variable but is usually poorer than other regions due to the low quality of grazing on infertile soils (Mandiringana et al., 2005; Gichangi et al., 2008). The CM is the most common feedstock for biogas production in the region, and the use of the BGS in agriculture, could contribute to soil fertility and offer a strategy for mitigating greenhouse gas emissions (Weiland, 2010). The process of biogas production from the CM could change the chemical composition of the feedstock and possibly make the nutrients more labile in the slurry. For example, Khan et al. (2012) reported that BGS contains higher N compared to the solid animal manure feedstock. However, Bonten et al. (2014) argued that the N, P, K contents of BGS and solid animal manure could be similar, if ammonia volatilization is prevented during anaerobic digestion. There is a need to understand the fertiliser value of BGS in sub-Saharan Africa, and how it compares with the feedstock (CM).

Many studies comparing BGS from other different organic sources and the feedstocks (solid manures) have been conducted (Paul and Beauchamp, 1993; Möller and Müller, 2012; Bonten et al., 2014). However, in some cases other waste streams are added during anaerobic digestion, which hampers the comparison between BGS and solid animal manure. Therefore, there is a paucity of studies that compare the fertiliser value of BGS and the feedstock (CM). Furthermore, most studies on BGS are conducted under controlled environmental conditions (i.e laboratory or glasshouses), with little research work under field conditions especially in sub-Saharan Africa, where temperature and soil moisture fluctuate. Application of BGS could help smallholder farmers in South Africa, who generally apply CF at less than the recommended rates because of the high cost. Maize is among the most commonly grown crops on the smallholder farms of South Africa (DAFF, 2016). The threshold for soil nutrient levels for maize are 8 mg NO_3_–N kg^−1^, 8.5 mg P kg^−1^, 80–125 mg K kg^−1^ (FSSA, 2007; Ayodele and Omotoso, 2008; Biljon et al., 2008). It was hypothesized that, when applied at the same rate based on N, BGS produced from CM from sub-Saharan Africa will result in the same maize dry matter, grain yield and nutrient uptake as the feedstoch (CM) and compound chemical fertiliser, with additional benefits of increasing available P under field conditions. The objective of this study was to determine the comparative effects of BGS and CM relative to CF on maize dry matter, grain yield and uptake of N, P and K of maize, and soil nutrient composition after crop harvest under field conditions.

## 2 Materials and methods

### 2.1 Site description

The study was conducted at the experimental station of the Agricultural Research Council-Vegetable and Ornamental Plants and the climatic and soil characteristics of the experimental site are as described by Mdlambuzi et al. (2021). The BGS and CM, experimental design and agronomic practices, including water management, are also as detailed in Mdlambuzi et al. (2021).

### 2.2 Plant sampling and analysis

Leaves of five randomly selected maize plants were sampled from each plot at the tasselling stage. The stems of the sampled plants were kept and included to the plants used for the determination of dry matter. All leaf samples were kept in well-labeled paper bags, oven-dried at 50 °C and ground to <0.5 mm using Fritsch Pulverisette mortar grinder. The ground plant samples were digested following the nitric acid-perchloric acid digestion method (Zasoski and Burau, 1977). Briefly, 0.5 g of dried plant material was digested with 7 ml nitric acid and 3 ml perchloric acid at 180 °C, brought up to volume in a 100 ml volumetric flask and analysed for P, K, Ca and Mg by inductively coupled plasma-optical emission spectroscopy (ICP-OES). Total N was determined by dry combustion method (Jimenez and Ladha, 1993). Maize grain yield was only determined in the 2016/2017 season because monkeys damaged the cobs just before harvest in the 2017/2018 season.

### 2.3 Selected physicochemical soil parameters after maize

After maize harvest, five soil subsamples were collected from the 0–20 cm depth of each plot using a bucket auger and mixed thoroughly to make a composite sample. The soil samples were analysed at the Agriculture Research Council's Soil, Climate and Water (ARC-SCW) laboratory. Total N was determined using the Kjeldahl digestion method (Bremner and Mulvaney, 1982). Plant available P was extracted with Bray 1 extraction solution (Bray and Kurtz, 1945) followed by analysis on a Continuous Flow Auto Analyser 3, *SEAL* Analytical, Australia. Exchangeable K was extracted with 1M Ammonium acetate solution (NH_4_OAc) adjusted to a pH of 7.0 (Chapman, 1965; Hesse, 1971) and analysed with an ICP (ICPES-9820, Plasma Atomic Emission Spectrometer, Shimadzu Corporation, Japan).

### 2.4 Statistical analysis

The data for maize dry matter, grain yield, uptake of N, P, K and soil concentration of N, P, and K were statistically analysed as described by Mdlambuzi et al. (2021). Maize dry matter yield was correlated with uptake of N, P, K and with soil concentrations of total N, available P, exchangeable K using Pearson's correlation analysis for each season using JMP statistical software (14^th^ edition).

## 3 Results

### 3.1 Dry matter and grain yield

**Figure 1:**
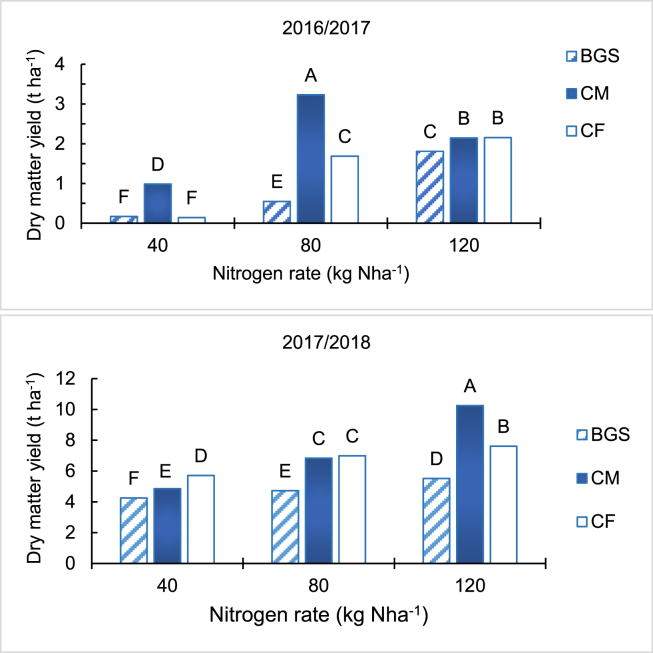
Effect of biogas slurry, cattle manure and chemical fertiliser on maize dry matter for the 2016/2017 and 2017/2018 planting seasons. Different letters indicate significant differences based on Tukey test at *p* < 0.01. 1 tonne per hectare equals 1000 kg per hectare.

Maize dry matter yield increased with increase in N rate for all the treatments (*p <* 0.01) for both seasons (Figure 1). In both seasons, the BGS treatment resulted in the lower dry matter than CM and CF at all N application rates except at 40 kg Nha^−1^ in the 2016/2017 season, where BGS was similar to CF. The CM had higher dry matter than CF at 40 and 80 kg Nha^−1^ but not at 120 kg Nha^−1^ where the two treatments had similar effects in the 2016/2017 season. In the 2017/2018 season the dry matter in the CF treatment was higher at 40 kg Nha^−1^, similar at 80 kg Nha^−1^ and lower at 120 kg Nha^−1^ than CM. The highest dry matter was in CM at 80 kg Nha^−1^ in the 2016/2017 season and at 120 kg Nha^−1^ in the 2017/2018 season followed by CF. The dry matter yield for all treatments was generally higher in the 2017/2018 than the 2016/2017 season.

**Figure 2:**
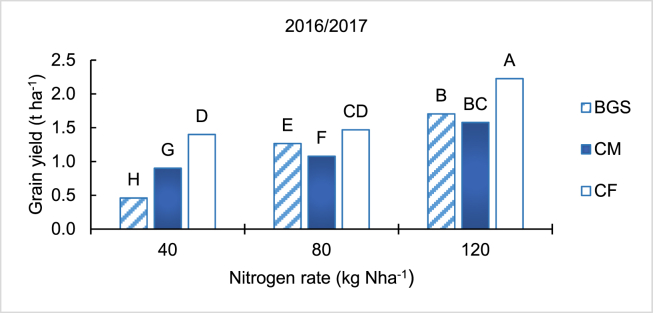
Grain yield of maize crop as influenced by different treatments and application rates for the 2016/2017 planting season. Different letters indicate significant differences based on Tukey test at *p* < 0.01.

Maize grain yield increased with increasing N rate for all treatments (*p <* 0.01) (Figure 2). The CF treatment had higher grain yield than both BGS and CM, at each rate in the 2016/2017 season. The grain yield in the BGS treatment was lower at 40 kg Nha^−1^, higher at 80 kg Nha^−1^, and similar at 120 kg Nha^−1^ when compared to CM.

### 3.2 Uptake of nitrogen, phosphorus and potassium

**Figure 3:**
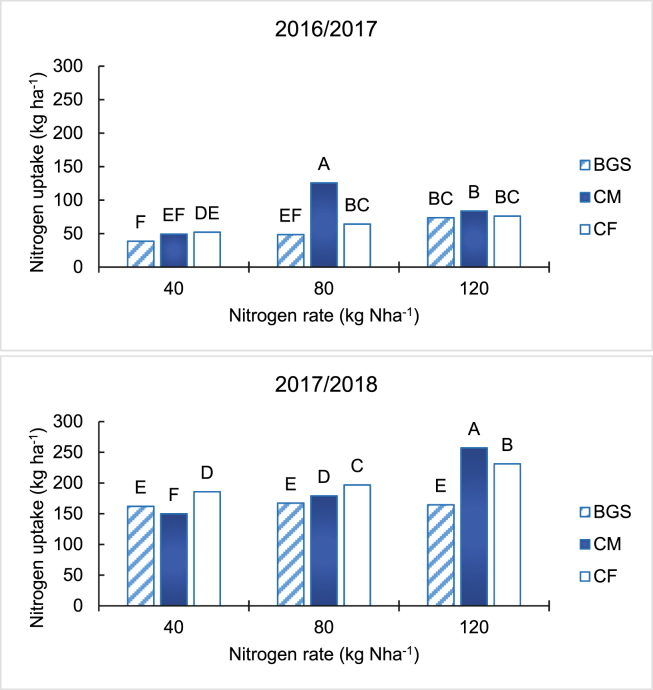
Effect of biogas slurry, cattle manure and chemical fertilizer on maize N uptake for the 2016/2017 and 2017/2018 planting seasons. Different letters indicate significant differences based on Tukey test at *p* < 0.01.

Increasing amendment rate increased N uptake by maize for all treatments (*p* < 0.01) in both seasons (Figure 3). The highest N uptake (higher than the other two) was from the CM treatment at 80 kg Nha^−1^ in the 2016/2017 and at 120 kg Nha^−1^ in the 2017/2018 season. In the 2016/2017 season, BGS had similar N uptake at 40 and 120 kg Nha^−1^ when compared with CM, which was similar to the CF treatment. In the 2017/2018 season, BGS resulted in lower N uptake than CF treatment at all N rates. The N uptake in the BGS treatment was higher at 40 kg Nha^−1^ and lower at 80 and 120 kg Nha^−1,^ than the CM treatment (Figure 3).

At each rate, the BGS treatment had lower P uptake than the CF and CM in both seasons except at 120kg Nha^−1^ in the 2016/2017 season, where BGS resulted in similar P uptake with CM. The highest P uptake was in 120 kg Nha^−1^ of the CF in the 2016/2017 season and of the CM treatment in the 2017/2018 season. In both seasons, higher rates increased K uptake except in the 2017/2018 season, where K uptake in the BGS treatment did not change (Table 1). The CF treatment resulted in higher K uptake than both BGS and CM in both seasons. BGS treatment had lower K uptake than CM in both seasons except at 120 kg Nha^−1^ in 2016/2017, where the two treatments were similar.

**Table 1:** Effect of increasing rate biogas slurry, cattle manure and chemical fertiliser (kg Nha^−1^) on uptake of phosphorus and potassium by maize.

Treatment	40	80	120
*Season 2016/2017*			
Phosphorus uptake (kg ha^−1^)			
BGS	0.69^E^	1.83^DE^	4.80^BC^
CM	3.99^BC^	5.88^AB^	4.49^BC^
CF	3.93^BC^	3.13^CD^	7.16^A^
Potassium uptake (kg ha^−1^)			
BGS	26.98^E^	37.69^D^	41.85^CD^
CM	37.92^D^	54.96^B^	46.29^C^
CF	46.43^C^	46.50^C^	63.37^A^
*Season 2017/2018*			
Phosphorus uptake (kg ha^−1^)			
BGS	13.44^E^	15.23^E^	18.92^D^
CM	20.94^CD^	20.90^CD^	37.80^A^
CF	22.62^BC^	22.13^C^	24.35^B^
Potassium uptake (kg ha^−1^)			
BGS	43.09^G^	41.81^GH^	38.35^H^
CM	54.59^F^	63.66^E^	144.5^A^
CF	100.2^D^	107.6^C^	117.6^B^

Different letters indicate significant differences based on Tukey test at *p* < 0.01.

### 3.3 Residual nutrients after maize

Increasing N application rate caused an increase in total N in the soil for all treatments in both seasons (Table 2). Total soil N in the BGS treatment was lower at 40 and 80 kg Nha^−1^ and higher at 120 kg Nha^−1^ than CM in the 2016/2017 season. In 2017/2018, BGS resulted in higher total N than CM treatment at all N application rates. At 40 and 80 kg Nha^−1^ BGS resulted in lower total N than CF with no difference at 120 kg Nha^−1^ in 2016/2017 season, while in the 2017/2018 season, BGS resulted into higher total N than CF treatment at 40 and 120 kg Nha^−1^ (Table 2).

**Table 2:** Effect of increasing rate of biogas slurry, cattle manure and chemical fertiliser (kg Nha^−1^) on total nitrogen, extractable phosphorus and exchangeable potassium in the soil after maize.

Treatment	40	80	120
*Season 2016/2017*			
*Total nitrogen (%)*			
BGS	0.02^D^	0.03^CD^	0.05^A^
CM	0.04^B^	0.04^B^	0.04^B^
CF	0.03^C^	0.04^B^	0.05^A^
Extractable phosphorus (mg kg^−1^)			
BGS	0.99^H^	6.07^E^	11.29^B^
CM	0.27^I^	2.97^G^	5.90^F^
CF	6.95^D^	8.60^C^	11.77^A^
Exchangeable potassium (cmol_c_ kg^−1^)			
BGS	0.07^G^	0.09^F^	0.10^E^
CM	0.11^D^	0.17^C^	0.24^A^
CF	0.20^B^	0.23^A^	0.24^A^
*Season 2017/2018*			
*Total nitrogen (%)*			
BGS	0.04^D^	0.05^C^	0.07^A^
CM	0.02^G^	0.03^F^	0.03^DE^
CF	0.03^EF^	0.05^C^	0.06^B^
Extractable phosphorus (mg kg^−1^)			
BGS	6.49^G^	14.64^B^	14.84^B^
CM	7.20^F^	9.30^E^	10.32^D^
CF	10.19^D^	11.44^C^	16.48^A^
Exchangeable potassium (cmol_c_ kg^−1^)			
BGS	0.14^I^	0.20^E^	0.19^F^
CM	0.16^H^	0.18^G^	0.31^D^
CF	0.33^C^	0.88^B^	1.07^A^

Different letters indicate significant differences based on Tukey test at *p* < 0.01.

The BGS treatment resulted in higher soil extractable P than CM at all N rates in both seasons except at 40 kg Nha^−1^ where CM had higher than BGS in the 2017/2018 season (Table 2). Extractable P in both BGS and CM treatments was lower than the CF treatment across all N application rates in both seasons, except at 80 kg Nha^−1^ in the 2017/2018 season where BGS had higher levels than CF treatment. The results showed lower exchangeable K in the BGS treatment than the CM treatment across all rates for both seasons except at 80 kg Nha^−1^ where BGS treatment had higher exchangeable K in 2017/2018 season. The CF treatment had higher exchangeable K than CM at all N rates for both seasons except at 120 kg Nha^−1^ in the 2016/2017 season (Table 2).

There was a strong positive correlation between maize dry matter and plant nutrient uptake for all three elements in both the 2016/2017 and 2017/2018 seasons (Table 3). Dry matter and soil concentrations of the different elements showed a weak positive correlation. Soil N and exchangeable K were the only elements that showed a slightly stronger positive correlation in the 2016/2017 season, while in the 2017/2018 season, only P showed a slightly strong positive correlation analysis (Table 3).

**Table 3:** Correlation analysis of dry matter with plant nutrient uptake and selected nutrients in the soil after maize.

	*Season 2016/2017*	*Season 2017/2018*
	Dry matter	Dry matter
Plant uptake		
Nitrogen	0.911	0.934
Phosphorus Potassium	0.716 0.694	0.931 0.871
Soil parameters		
Total nitrogen	0.577	-0.032
Available phosphorus	0.259	0.199
Exchangeable potassium	0.473	0.418

## 4 Discussion

The increase of maize dry matter and grain yield with the increasing rate of the amendments could be explained by higher uptake of N (Figure 3), K and to a lesser extent P (Table 1) in both seasons. The higher nutrients increased maize growth and development, leading to higher dry matter yields (Islam et al., 2010). This view was supported by the strong positive correlation between N uptake and dry matter yield in both seasons. The dry matter yields results of our study agreed with Rahman et al. (2008), who indicated that higher cattle slurry rates (kg Nha^−1^), increased maize dry matter, as a result of increased vegetative growth. The higher biomass accumulation was a result of increased N availability and uptake of other nutrients. This is supported by the strong positive correlation between dry matter yield and uptake of P and K in both 2016/2017 and 2017/2028 seasons for all the treatments (Table 3). The supply of nutrients by the amendments, resulted in their greater uptake and growth of maize, especially at higher N application rates. The lower dry matter yield in the BGS than CM and CF treatments was in response to lower uptake of N (Figure 3), P and K (Table 1) in both seasons. Nutrients in BGS are higher than in CM, which agreed with Möller and Müller (2012). The BGS in this study had high N (2.55%) and K (1.77%) and low P (0.57%) compared to the ranges found in the literature (0.5–2.5% N and 0.5–1.5% P, 0.6–2.2% K). The CM had higher N (1.9%), lower P (0.337%), and high K (1.67% K) than values in the literature where manure was used for BGS production (0.4–0.8% N, 0.6–0.8% P and 0.5–0.7% K). The higher N and K and the lower P in the manure translated into the same trend in the composition of the BGS. The lower P could be a result of low geological reserves (Mandiringana et al., 2005) and fixation to iron and aluminium oxides (Gichangi et al., 2008) in tropical and subtropical soils of sub-Saharan Africa, resulting low uptake by the plants (grass) on which the cattle fed on. When the BGS and CM are applied at the same rate of N, any differences between the two treatments are a result of differences in availability of the N and concentrations of other nutrients. The digestion process (biogas production) is believed to convert organically bound nutrients into more readily available forms (Bonten et al., 2014). However, we observed lower dry matter yield in the BGS treatment than in the CM, especially in the first season suggesting that this effect was not evident. The CM supplied significantly more K than the BGS treatment, when applied at the same N rate, which could explain the higher dry matter in CM. This view was supported by higher K uptake in CM than BGS in both seasons. The higher K uptake in the CF, than the CM, is explained by the more readily available N in the CF, which increased biomass accumulation.

The generally lower maize nutrient uptake and dry matter in the 2016/2017 than the 2017/2018 was explained by late planting resulting in a shorter growing season. The maize was planted in mid-January in the first and in mid-December in the second season. Likewise, the lower grain yield determined (3.4 t ha^−1^) than expected in the CF treatment (5 t ha^−1^) at 120 kg N ha^−1^ was explained by the late planting (short season) in the 2016/2017 season from which the grain yield data were collected. Like dry matter, the increase in grain yield with an increase in N rates could be explained by increased available N, P and K, which increased their uptake and growth, including grain filling. These results agreed with results reported by Henson and Bliss (1991), Yu et al. (2010) and Malav et al. (2015b). The similarity in the trends between dry matter and grain yield, suggested that higher dry matter resulted in higher grain yield for maize. While the maize cobs were damaged by monkeys in the 2017/2018 season, the higher dry matter, than in the previous season, suggested that higher grain yield could have been realised in the second season. However, the higher grain yield in the CF treatment than both the BGS and CM, even though the dry matter was lower than CM in the 2016/2017 season (only studied), was a result of higher uptake of P and K, especially at higher N rate (120 kg Nha^−1^).

The organic sources (BGS and CM) released nutrients at a slower rate than CF, which explained the lower grain yield from both BGS and CM treatments (Xu et al., 2019). The grain yield in the BGS treatment, which was higher at 40 kg Nha^−1^, lower at 80 kg Nha^−1^ and similar at 120 kg Nha^−1^, when compared to CM, indicated that the N fertiliser value of the two materials was equivalent, especially after correcting for P. Achieving similar yield between BGS and CM indicates that there is greater benefit in using CM to produce biogas (energy) followed by using the resultant BGS as an organic fertiliser. It needs to be noted that without additional P as single superphosphate, the benefits of the BGS, together with the CM, would have been limited by low availability of P. Additional P is required if significant yield benefits are to be derived from BGS originating from CM in sub-Saharan Africa. The benefits of the treatments (BGS, CM and CF) were dose dependant as seen from the different plant nutrient uptake and soil nutrient reserves results (Tables 1, 2, and 3). While BGS gave lower yields than CF, its use as a basal fertiliser, with top dressing with mineral fertiliser, may increase yields while improving overall soil quality in low input smallholder agroecosystems in sub-Saharan Africa where similar soil and manure quality occur.

The higher total N, extractable P and exchangeable K in soil with the increasing rate was due to higher additions through the amendments. Abubaker et al. (2013), Bonten et al. (2014) and Xu et al. (2019) showed that the application of BGS and manure influenced nutrients in soil. The slower release of nutrients by BGS, as for CM, could be beneficial in meeting the nutritional requirements of the subsequent crop (Bharde et al., 2003). The higher extractable P, for both seasons, from the BGS treatment than the CM treatment could be attributed to the higher organic P added as BGS and lower uptake by maize. Although the P was corrected with straight fertiliser, the bulk in the BGS and CM was in organic form and needed to be mineralised, while higher amounts added as superphosphate to the CM (correction) than BGS resulted in greater uptake. While Tarkalson and Leyten (2009) argued that soils treated with either liquid or solid CM resulted in higher availability of P, the results of this study showed higher soil available P in CF than the organic sources. The BGS resulted in higher P than CM but both BGS and CM treatments were lower than the CF treatment in both seasons except at 120 kg Nha^−1^ in 2017/2018 season. The higher soil nutrients observed after harvest in the second than the first season of planting for all treatments was a cumulative effect of the two years of application together with limited uptake in the first season, due to the lower biomass accumulation as a result of late planting. In the CF treatment, all the P was added in readily available form, while only a portion (correction using straight fertiliser) in the BGS and CM were readily available, with the rest requiring mineralisation. Zhang et al. (2006) indicated that the release of nutrients from organic waste sources, including manure, mostly occurs in the second season after application. The higher soil P in the BGS treatment than CM was a result of lower uptake by maize (Table 1), and higher pH (Mdlambuzi et al., 2021), which could have reduced fixation, making P more readily available. These benefits are in addition to the increased soil organic C reported in Mdlambuzi et al. (2021).

## 5 Conclusions

The study demonstrated that application of BGS to agricultural soils provided available N, P and K, which supported maize growth and built up soil reserves. The increase in dry matter and grain yield (first season) from BGS and CM was, however, lower compared to the N:P:K (3:2:1 (28)), when applied at the same rate of N. While BGS application resulted in lower uptake of N, P and K than CM, the soil under this treatment had higher available P. The relatively higher dry matter, nutrient uptake and soil nutrients after harvest of maize fertilised by BGS, and CM, showed cumulative effects of repeated application. The study has improved our understanding of the fertiliser value of BGS and its feedstock (CM) produced in sub-Saharan Africa on maize and has provided basis for research on the use of BGS as a source of nutrients for crop production especially for smallholder farmers. Where CF is not readily accessible, farmers could use CM to produce biogas and use the secondary product (BGS) with the same or even better nutrient value with the CM to improve soil fertility and crop yields. In the first season, BGS may need to be applied at double the rate of CF to achieve similar dry matter and grain yield. Further research should focus on the co-application of BGS with CF on crop productivity, soil quality and carbon dioxide emissions from agricultural soils.

## Declarations

### Author contribution statement

Mdlambuzi T.: Conceived and designed the experiments; Performed the experiments; Analyzed and interpreted the data; Wrote the paper.

Muchaonyerwa P.; Tsubo, M.: Conceived and designed the experiments; Analyzed and interpreted the data; Wrote the paper.

Moshia M.E.: Analyzed and interpreted the data; Wrote the paper.

### Funding statement

This work was supported by the 10.13039/501100001321National Research Foundation(Grant UID 97841).

### Data availability statement

Data will be made available on request.

### Declaration of interests statement

The authors declare no conflict of interest.

### Additional information

No additional information is available for this paper.
